# Fournier’s gangrene in a rectal cancer patient

**DOI:** 10.1016/j.ijscr.2020.01.040

**Published:** 2020-02-06

**Authors:** Dong woo Hyun, Byoung Chul Lee, Jung Bum Choi, Young Mok Park, Hyuk Jae Jung, Hong Jae Jo

**Affiliations:** Department of Surgery, Pusan National University Hospital, 179, Gudeok-ro, Seo-gu, Busan 49241, South Korea

**Keywords:** Fournier’s gangrene, Rectal cancer, Necrotizing fasciitis

## Abstract

**Introduction:**

Fournier’s gangrene (FG) is caused by a variety of causes, but FG led by rectal cancer is rare.

**Presentation of case:**

A 62-year-old man presented with perineal pain for several days. Multiple black spots on the scrotum and perineum surrounded by erythema were found on a physical exam. Computed tomography showed diffuse air density with subcutaneous edema in the perineum, scrotum, anus, and left lower abdominal wall and showed 4.1 cm-size mass in anus. He was diagnosed with FG caused by rectal cancer. He underwent extensive debridement of the perineum, scrotum, medial buttocks, and diverting loop colostomy of transverse colon. After repeated debridement of some residual necrotic tissue, abdominal perineal resection was performed after 24 days after initial surgery. The patient received reconstruction surgery of the soft tissue defect and discharged on postoperative day 84. He is being followed up without any recurrence for 10 months.

**Conclusion:**

Prompt clinical diagnosis and urgent surgical management are crucial for patient’s favorable outcome. The patient in our case study could be recovered by a combined modality therapy we provided.

## Highlights

• Rectal cancer induced Fournier's gangrene is mainly caused by cancer perforation.
• Vacuum assisted closure treatment helps to accelerate the healing of surgical wound.
• Prompt diagnosis and urgent surgery are crucial for patient’s favorable outcome.

## 1 Introduction

Fournier’s gangrene (FG) is a necrotizing fasciitis occurring and rapidly and progressively expanding in the external genitalia and the perineum. FG is caused by a variety of causes, but FG led by rectal cancer is rare. A specific risk factor is rectal cancer perforation. Rectal cancer-induced FG usually behaves more aggressively and is associated with higher mortality than FG from other causes [1]. FG requires rapid diagnosis and urgent surgical debridement and in the setting of rectal cancer, tumor resection is necessarily performed.

We report an unusual case of severe FG caused by locally advanced rectal cancer and provide a combined modality therapy. This case report has been reported in line with the surgical case report (SCARE) criteria [2].

## 2 Presentation of case

**Fig. 1:**
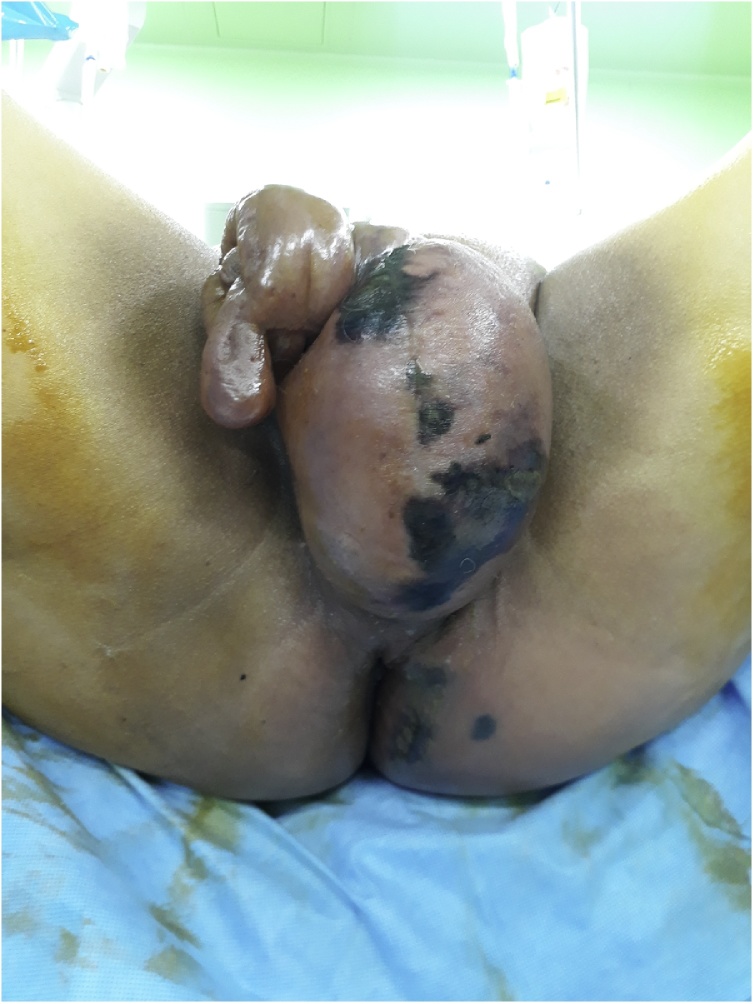
Infected perineum of the patient. Multiple black spots on the scrotum and perineum surrounded by erythema.

**Fig. 2:**
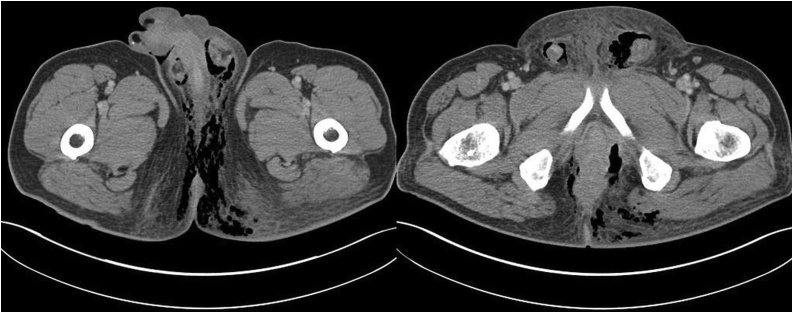
Axial computed tomography image of abdomen and pelvis showing rectal tumor with necrotizing fasciitis in perineal area.

A 62-year-old male patient visited our emergency room with perineal pain which had been presented for several days. He complained of swelling and pain of perineum with general fatigue. The medical history was unremarkable. A physical examination revealed multiple black spots on the scrotum and perineum surrounded by erythema (Fig. 1). Digital rectal examination revealed a circumferential bulging mass located in the low rectum. The patient’s blood pressure was 130/80 mmHg, pulse rate was 130 beats/min, and body temperature was 36.7 °C. Electrocardiogram showed sinus tachycardia. In the complete blood count, a white blood cell count was 27.26 × 10^3^/mm^3^ and hemoglobin was 11.0 g/dL. The C-reactive protein was 37.72 mg/dL, Procalcitonin was 36.61 ng/mL, Creatinine was 5.17 mg/dL, GFR was 11.4 mL/min, fasting glucose level was 209 mg/dL, HbA1c was 7.6 %, Potassium was 4.5 mmol/L, and the carcinoembryonic antigen was 4.3 ng/mL. He underwent computed tomography (CT), which showed diffuse air density with subcutaneous edema in the perineum, scrotum, anus, and left lower abdominal wall and showed 4.1 cm-size mass in anus (Fig. 2). The patient was diagnosed with Fournier’s gangrene caused by locally advanced rectal cancer.

**Fig. 3:**
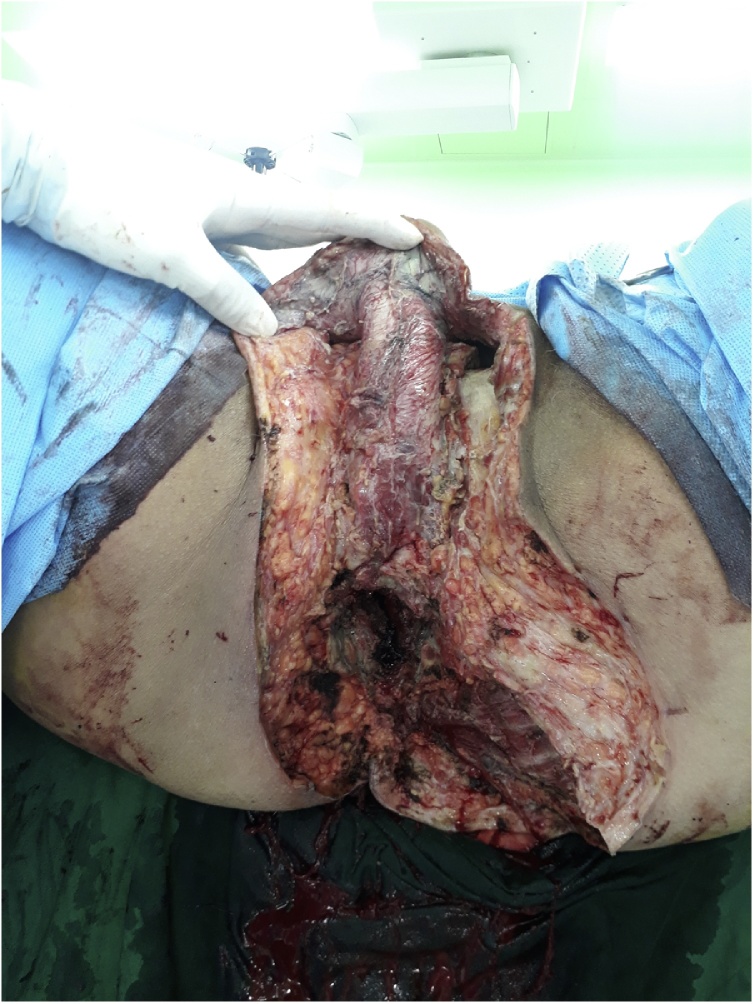
Extensive debridement of the perineum, scrotum, medial buttocks was performed.

The patient underwent urgent extensive debridement of the perineum, scrotum, medial buttocks and diverting loop colostomy of transverse colon in December 2018 (Fig. 3). Operative findings revealed extensive necrotic tissues in perineum, scrotum, and perianal area with solid cancerous mass arising from lower rectum. Biopsy revealed a moderately differentiated adenocarcinoma. The patient was taken back to the operating room 2 days later for a repeated debridement of residual necrotic tissue. He received treatment for septic shock and nutritional support in the intensive care unit. His recovery was remarkable and was transferred to the general ward on postoperative day 10. After repeated debridement of some residual necrotic tissue, abdominal perineal resection was performed after 24 days after initial surgery. The pathology results revealed a T3N0 moderate adenocarcinoma, negative resection margins, and positive lymphovascular and perineural invasion. CT revealed no metastases in other organs.

**Fig. 4:**
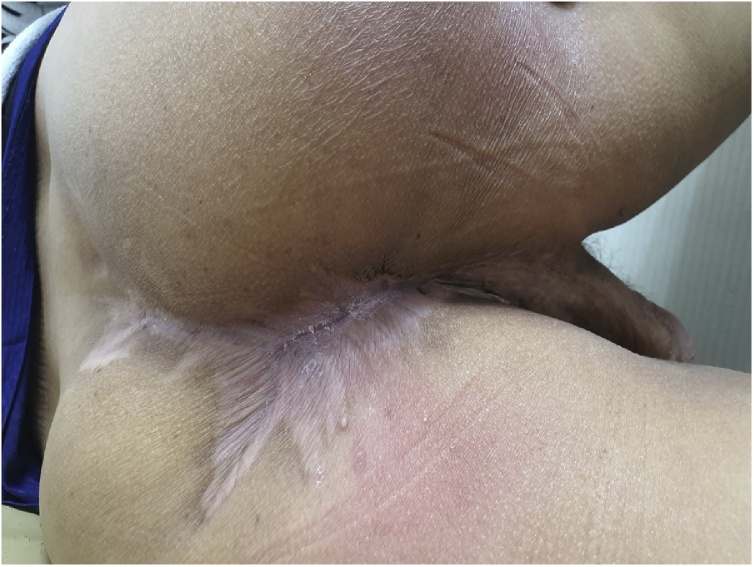
Final appearance of complete wound recovery.

The perineal wound was treated by applying a CuraVAC® (Negative pressure wound therapy) and daily dressing. After the perineal wound stabilized, he received reconstruction surgery of the soft tissue defect by plastic surgery team (Fig. 4). There were no major postoperative complications, and he was discharged on postoperative day 84. He did not receive chemoradiotherapy because of wound management. He is being followed up without any recurrence for 10 months.

The Institutional Review Board of Pusan National University Hospital approved this study and waived the informed consent requirement.

## 3 Discussion

Fournier gangrene (FG) is a form of necrotizing fasciitis that affects the genitals, perineal and perianal region resulting from a polymicrobial infection whose source can be genitourinary, colorectal, skin or idiopathic, which could be potentially lethal [3]. The predisposing factors associated with FG are diabetes, alcoholism, immunosuppression, cytotoxic drugs, chronic steroid use, smoking, lymphoproliferative diseases, malnutrition, and HIV infection [3,4]. Its etiology is identified in 75–100 % of patients. Its origin is colorectal in 13–50 % of cases, urogenital in 17–87 % of patients [5,6]. Other causes include skin infections and local trauma. Colorectal sources include perirectal and perianal abscess, rectal instrumentation, large bowel perforation due to colon cancer, diverticulitis, hemorrhoids, and anal intercourse among homosexuals [[7], [8], [9]].

The incidence of rectal cancer-induced FG in all-cause FG ranged from 1.47 % to 16.6 %. A specific risk factor of rectal cancer-induced FG is rectal cancer perforation [1]. Although perforation of rectal cancer after treatment with bevacizumab or radiation therapy has been well documented, reports on spontaneous perforation of rectal cancer presenting as FG are rare [10]. There were 40 cases of FG caused by spontaneous perforation of rectal cancer. 23 cases were evaluated in a review by Bruketa et al. and 17 cases have been reported by Yoshino et al. [1,10]. Diabetes mellitus was considered a most common risk factor. We had a patient with diabetes mellitus in our case study. Treatments for rectal cancer with FG include abdominoperineal resection, extralevator abdominoperineal resection, and total pelvic exenteration. Reconstruction surgery is considered when an extensive healthy granulation tissue formation on the wound base is present.

FG is life-threatening and has an extremely high mortality rate. Therefore, early diagnosis and aggressive management are crucial for patient’s outcome. Clinical diagnosis becomes evident when there is edema, crepitus, and areas of dark red color moving rapidly towards extensive gangrene. Computed tomography (CT) is the most effective tool for diagnosing FG, identifying the infectious origin, and delineating the extent of the disease [11]. Soft tissue thickening, inflammation, and subcutaneous emphysema are the CT features found in FG. Aggressive debridement, broad-spectrum antibiotics, and intensive supportive care are critical for the management of FG. The initial debridement with adequate resection of the non-viable tissues is considered the most important factor for survival. All non‐viable and necrotic tissue must be excised until well perfused viable tissue is reached [12]. In the case of uncontrolled infections and necrosis, repeated surgical debridement should be done. Colostomy is needed to prevent fecal contamination in cases of severe perineal involvement in all-cause FG. Vacuum Assisted Closure (VAC) treatment is a method employed to accelerate the healing of surgical wounds and complicated wounds that fail primary healing [13]. Patient with FG may need multiple debridement surgeries, often resulting in significant soft-tissue loss requiring reconstruction.

## 4 Conclusion

In conclusion, prompt clinical diagnosis and urgent surgical management are crucial for patient’s favorable outcome. The patient in our case study could be recovered by a combined modality therapy we provided.

## Funding

This work was supported by a 2-year Research Grant of Pusan National University.

## Ethical approval

The Institutional Review Board of Pusan National University Hospital approved this study and waived the informed consent requirement.

## Consent

Written informed consent was obtained for publication of this case report and accompanying images. A copy of the written consent is available for review by the Editor-in-Chief of this journal on request.

## Author contribution

Dong woo Hyun, Jung Bum Choi – writing, original drafting.

Young Mok Park, Hyuk Jae Jung – review and editing.

Hong Jae Jo – funding acquisition, revision.

Byoung Chul Lee – supervision/corresponding author.

## Registration of research studies

The authors declare that no registration is needed for this work.

## Guarantor

Byoung Chul Lee, MD.

## Provenance and peer review

Not commissioned, externally peer-reviewed.

## Declaration of Competing Interest

The author has no conflict of interest to declare.
